# Helix-Aggregation Interplay in Nucleophosmin 1: Structural, Morphological, and Cytotoxic Consequences of Fragment Length

**DOI:** 10.1021/acsomega.6c03067

**Published:** 2026-05-13

**Authors:** Daniele Florio, Ilaria Leone, Sara La Manna, Alessia Cugudda, Flavia Anna Mercurio, Marilisa Leone, Daniela Marasco

**Affiliations:** † IRCCS SYNLAB SDN, via G. Ferraris 144, 80146 Naples, Italy; ‡ Department of Pharmacy, School of Medicine and Surgery, University of Naples Federico II, Via Domenico Montesano 49, 80131 Naples, Italy; § Institute of Biostructures and Bioimaging (IBB), CNR, Via P. Castellino 111, 80131 Naples, Italy

## Abstract

Nucleophosmin 1 (NPM1) undergoes liquid–liquid phase separation (LLPS) and can form amyloid-like aggregates when structural elements within its C-terminal domain become destabilized. The helical region spanning residues 264–277 represents a key amyloidogenic segment, and its conformational balance is involved in NPM1 misfolding in acute myeloid leukemia (AML). Here, we examined how N- and C-terminal sequence extensions modulate the structural and aggregative behavior of two C-terminal fragments, NPM1_259–280_ and NPM1_263–280_. Although the two fragments exhibit comparable theoretical charge properties, ThT kinetics revealed markedly different aggregation profiles, with the shorter NPM1_263–280_ fragment aggregating more rapidly and to a greater extent. Circular dichroism, Fourier transform infrared (FT-IR), and NMR analyses demonstrated that both peptides possess an intrinsic α-helical propensity, particularly across residues 267–273, and that stabilization of this helical population by 2,2,2-trifluoroethanol (TFE) reduces β-sheet formation and delays aggregation. Electron microscopy confirmed that the two fragments form fibrils with distinct morphologies and that TFE alters fibril organization by promoting more bundled architectures. Functionally, both peptides reduced OCI-AML2 cell viability, with NPM1_259–280_ showing the strongest effect; however, helix-stabilizing conditions reduced cytotoxicity. Together, these findings demonstrate that N-terminal extension exerts a protective effect on the aggregation-prone 264–277 region by stabilizing local α-helical structure and delaying the α → β transition that underlies NPM1 aggregation, thereby attenuating fibril formation and cytotoxicity. These results suggest that helical stabilization could limit the aggregation propensity of C-terminal regions of NPM1 and exert a potential protective mechanism against its misfolding.

## Graphical abstract

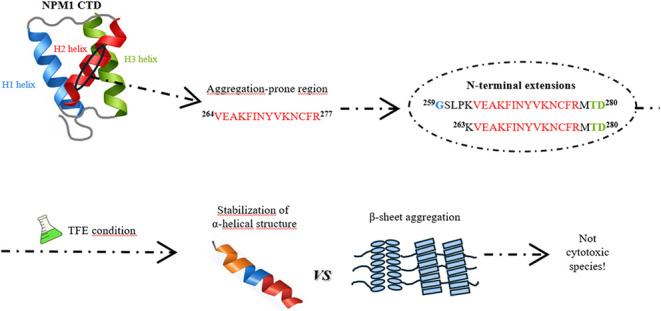

## Introduction

The amyloid hypothesis proposes that soluble oligomers, common to most amyloid-forming proteins, represent the primary toxic species involved in disease pathology.^1^ These oligomers share a conformation-dependent structural β-motif that is conserved across different amino acid sequences.^2^ More recently, an alternative amyloid formation route, termed “the condensation pathway”, has been described.^3^ In this mechanism, proteins first undergo liquid–liquid phase separation (LLPS) before aggregating into amyloid fibrils.^4^ LLPS involves the demixing of a homogeneous protein solution into coexisting liquid phases of different densities.^5,6^ Within the condensed phase, high local protein concentrations significantly accelerate aggregation processes. This mechanism has been observed for several neurodegeneration-related proteins, including α-synuclein, tau, and FUS.^7–9^ Recent studies have further clarified the link between LLPS and amyloid formation. LLPS has been proposed to act as a nucleation mechanism that promotes protein misfolding and amyloid aggregation in neurodegenerative disease models.^10^ In amyloid-β, phase-separated droplets under crowded conditions can serve as nucleation sites that modulate aggregation kinetics and fibril formation.^11^ LLPS of α-synuclein has been shown to produce structurally diverse fibrils with altered toxicity, indicating that the droplet environment influences both morphology and biological activity.^12^ Phase separation is also relevant for tau and Aβ in Alzheimer’s disease (AD), affecting aggregate assembly and potentially contributing to disease progression.^13,14^ Amyloid fibrils from diverse proteins such as Aβ, α-synuclein, prion protein, and transthyretin share a conserved cross-β architecture, characterized by β-strands oriented perpendicular to the fibril axis and stabilized by extensive interstrand hydrogen bonding.^15–17^ In many systems, amyloidogenesis involves a structural transition from α-helical or disordered conformations to β-sheet-rich structures (the α → β transition). Partial unfolding or destabilization of α-helical segments exposes aggregation-prone regions that convert into β-strands, enabling intermolecular hydrogen bonding and aggregation.^18^ In Aβ polypeptide, implicated in AD, transient α-helical regions (residues 15–24 and 30–36) unfold and reorganize into β-strand-rich oligomers that act as aggregation-prone sites.^19^ Stabilizing these helices—via mutations or ligand binding—delays aggregation, suggesting a protective role for α-helical structure.^20,21^ α-synuclein, involved in Parkinson’s disease (PD), is a protein known to adopt disordered, helical, and β-conformations.^22^ In its membrane-bound state, α-synuclein exhibits partial α-helicity, but denaturation or membrane detachment triggers α → β transitions in hydrophobic segments, promoting β-rich oligomerization and fibrillization.^23^ Phase-transition models propose that amyloid aggregation is governed by supersaturation and solubility thresholds, with α → β conversion acting to overcome nucleation barriers. Spectroscopic studies consistently show α-helix loss and β-sheet formation during aggregation, including characteristic circular dichroism (CD) shifts (222 vs 216 nm) and Fourier transform infrared (FTIR) amide I changes. Environmental factors (pH, ionic strength, temperature, interfaces) and sequence features (hydrophobic/polar patterning, aromatic residues, β-promoting motifs) further modulate these conformational transitions.

Nucleophosmin 1 (NPM1, UniProt P06748), an abundant nucleolar protein, forms large oligomers and undergoes LLPS through interactions among its modular domains. Its N-terminal β-barrel (residues 1–120) forms pentamers and decamers,^24^ the central intrinsically disordered region (residues 120–240) mediates multivalent interactions driving LLPS,^25^ and the C-terminal three-helix bundle (residues 240–294) binds nucleic acids.^26^ Acute myeloid leukemia (AML)-associated mutations in the C-terminal domain disrupt RNA binding, introduce a nuclear export signal, and promote aggregation.^27^ Biophysical studies have shown that wild-type NPM1 can form dynamic, reversible liquid-like condensates *in vitro*, driven by multivalent interactions and phase separation mechanisms.^28,29^ Under altered RNA-binding conditions or in certain mutant contexts, NPM1 assemblies exhibit increased viscosity and altered structural features, suggestive of more stable aggregate states. Recent cellular studies have also reported aberrant aggregate formation associated with NPM1 mutations.^30^ The disordered acidic region interacts with the C-terminal domain to cross-link pentamers via a “grappling-hook” mechanism, modulating NPM1’s chaperone-like activity against amyloid formation. Destabilization of the second and third helices within the C-terminal domain initiates β-sheet fibril formation and cytotoxic assembly formation, effects that are enhanced by disease-associated mutations.^31^

In recent years, we have extensively investigated the conformational effects of AML-associated mutations in the C-terminal domain,^12^ focusing on the native three-helix bundle^32,33^ —particularly its third helix (H3)^34–36^ —as well as on adjacent regions, including H2 (residues 264–277)^37–39^ and H1.^40^ Our working hypothesis is that AML mutations disrupt the native three-helix bundle, thereby exposing the highly amyloidogenic segment 264–277, which may trigger amyloid aggregation of the full-length NPMc+ protein.

In a previous study, we suggested that simultaneous N- and C-terminal extensions of the aggregation-prone segment NPM1_264–277_ markedly delayed aggregation and preserved mixed secondary structure even after 24 h, with a cooperative protective effect against β-sheet aggregation.^41^

This study is a comparative biophysical analysis of how minimal N-terminal extensions, concerning residues of the loop connecting H1 with H2, modulate aggregation kinetics, secondary structure propensities, and aggregate morphology of the NPM1 C-terminal fragment of the 264–277 region.

We investigated the conformational and aggregation behaviors of two NPM1 fragments, 259–280 and 263–280, in the presence and absence of 2,2,2-trifluoroethanol (TFE), using spectroscopic and microscopic techniques. TFE was employed as a helix-promoting cosolvent to probe the intrinsic secondary structure propensities of the peptides and their relationship with aggregation behavior. While TFE does not mimic physiological conditions, it is widely used to stabilize transient α-helical conformations in aggregation-prone peptides, enabling assessment of how helix stabilization correlates with aggregation delay rather than complete inhibition.^42^

Additionally, we assessed the cytotoxic effects of these two regions in leukemic OCI-AML2 cells.

## Materials and Methods

### Peptide Synthesis

The NPM1_259–280_ and NPM1_263–280_ peptides were synthesized through solid-state peptide synthesis (SSPS) as already reported^34^ and purified by high-performance liquid chromatography (HPLC) to >99% purity. After purification, the peptides were treated with 1,1,1,3,3,3-hexafluoro-2-propanol (HFIP, 50% v/v), the solvent was removed under a gentle nitrogen flux, and the peptides were lyophilized and stored at −20 °C until use.

The isoelectric point and net charge were calculated using the PepCalc tool (Innovagen, https://pepcalc.com).

Aggregation experiments were performed at 25 °C under continuous shaking at 400 rpm. Buffer composition and concentration were assay-dependent and are specified in the corresponding sections.

### Fluorescence Assays

The ThT fluorescence emission assay was performed in black 96-well plates under stirring using a fluorescence reader (Envision 2105, PerkinElmer), equipped with orbital shaking (400 rpm), at 25 °C using a ThT concentration of 50 μM and 50 mM phosphate buffer (pH = 7.4). Peptides were assayed at a concentration of 200 μM both in the absence and in the presence of 2,2,2-trifluoroethanol (TFE) (50% v/v). The half-time (*t*_1/2_) values were determined by nonlinear regression analysis of fluorescence emission versus time data using GraphPad Prism 8 (GraphPad Software, San Diego, CA), fitting the data to a Hill equation^43^ as follows:

$$F\left(t\right)=F_{\max}\frac{{\left(t/t_{1/2}\right)}^{n}}{1+{\left(t/t_{1/2}\right)}^{n}}$$

where *F*(*t*) is the fluorescence intensity at time *t*, *F*_max_ is the maximum fluorescence intensity, and *n* is a cooperativity parameter (*n* = 1, noncooperative model).

### Circular Dichroism

CD spectra were registered in the far-UV region from 190 to 260 nm on a J-810 spectropolarimeter (JASCO Corp., Milan, Italy) at a concentration of 200 μM in 20 mM phosphate buffer pH 7.4, both in the absence and in the presence of TFE (50% v/v) and stirred with Multi Reax rotator. Measurements were performed in a 0.1 cm path length cuvette at 25 °C with the following settings: scan speed of 20 nm/min, bandwidth of 2.0 nm, resolution of 0.2 nm, sensitivity of 50 mdeg, and response time of 4 s. Deconvolutions of CD spectra were obtained by BESTSEL software (http://bestsel.elte.hu/).^44^

### FTIR Spectroscopy

FTIR spectra were recorded in transmission mode at a peptide concentration of 300 μM, 50 mM phosphate buffer (pH 7.4), both in the absence and presence of TFE (50% v/v), using a Jasco FT/IR 4100 spectrometer (Easton, MD). Prior to analysis, all samples were stirred for 15 min to promote aggregation. For samples containing TFE, the solvent was first evaporated under a nitrogen flux. Subsequently, all samples were dried under vacuum to remove residual solvents before measurement. The measurements were performed with a Ge single crystal at a resolution of 4 cm^–1^. A total of 100 scans were recorded for each sample at a rate of 2 mm/s against a KBr background. After the collection in transmission mode, the spectra were converted to emission. Amide I deconvolutions were automatically returned as emissions with the built-in software (Spectra Manager 2.5). Deconvoluted amide I band frequencies and secondary structure assignments for the peptides alone and in the presence of TFE (50% v/v) were obtained by evaluating the second-derivative spectrum, which was calculated using a 7-point Savitsky–Golay second-derivative function.^45^

### NMR

NMR conformational analyses were performed at two concentrations (640 and 200 μM) in a mixture composed of 10 mM sodium phosphate buffer pH 7.4 and 2,2,2-trifluoroethanol-*d*_3_ (TFE-*d*_3_, 99.5% isotopic purity, Sigma-Aldrich by Merck Group, Milan, Italy) (50:50, v/v) at *T* = 25 °C. One-dimensional (1D) [^1^H], two-dimensional (2D) [^1^H, ^1^H] total correlation spectroscopy (TOCSY) (70 ms mixing time),^46^ and nuclear overhauser enhancement spectroscopy (NOESY) (300 ms mixing time)^47^ experiments were registered on a Bruker Avance 500 MHz spectrometer provided with a cryoprobe. NMR experiments were recorded with 16–32 scans, 128–180 free induction decay (FIDs) in t1, 1024, or 2048 data points in t2. Water suppression was obtained by Excitation Sculpting,^48^ and proton resonance assignments were obtained for peptides at a concentration equal to 640 μM by comparison of TOCSY and NOESY spectra and a standard protocol;^49^ peptide chemical shifts were referenced to the water signal (4.75 ppm). NMR spectra were processed with the software Bruker TopSpin 4.3.0 (Bruker, Milan, Italy) and analyzed with the software NEASY^50^ included in CARA (Computer Aided Resonance Assignment) (http://cara.nmr.ch/doku.php/). Chemical shift deviations from random-coil values for Hα protons (δΔHα) were calculated through the method suggested by Kjaergaard et al. (https://www1.bio.ku.dk/english/research/bms/sbinlab/randomchemicalshifts1 ),^51^ random-coil Hα chemical shift reference values were estimated at *T* = 25 °C and pH = 7.4. Based on DdHa values, the percentage of helical content was estimated with the following equation: [DdHa_ave_/(−0.39)] × 100, where DdHa_ave_ refers to mean values calculated considering only residues associated with negative Δδ_Hα_ and consequently helical structuration.^52^

### Scanning Electron Microscopy (SEM) Analysis

Field-emission SEM (Phenom_XL, Alfatest, Milan, Italy) was used to evaluate the morphologies of aggregates of peptides (200 μM) in the absence and in the presence of TFE (50% v/v), after 4 h of stirring. Then, ∼30 μL of the solution was drop-cast on an aluminum stub and dried under a vacuum to prepare the samples. For 75 s, a thin layer (∼7 nm) of gold was sputtered at a current of 25 mA. Following the introduction of the sputter-coated samples into the specimen chamber, micrographs were taken using a secondary electron detector (SED) at an accelerating voltage range of 10–15 kV.

### Cells

Human acute myeloid leukemia cell line OCI-AML2 was from IRCCS Synlab SDN Biobank^53^ and cultured according to the manufacturer’s recommendations. Briefly, cells were grown at 37 °C in a humidified atmosphere of 5% CO_2_ in RPMI 1640 (GIBCO, # 11875093) supplemented with 20% fetal bovine serum (FBS), 100 U/mL penicillin, 100 mg/mL streptomycin, and 1% l-glutamine. Then, cells were seeded in triplicate in 96-well plates at a density of 8.000 cells/well and allowed to adhere overnight.

### (3-[4,5-Dimethylthiazol-2-yl]-2,5 Diphenyl tetrazolium bromide) (MTT) Assay

NPM1_259–280_ and NPM1_263–280_ peptides in the absence and in the presence of 50% TFE (stock solution 800 μM in 50 mM phosphate buffer at pH 7.4), after 0 and 4 h of stirring, were diluted in a cell culture medium at a final concentration of 200 μM. Prior dilution for TFE-containing samples, the organic solvent was removed under vacuum. Dilute samples were added to the cells for 24 h. The control was constituted by untreated cells. After incubation, MTT assays were used according to the manufacturer’s instructions. Briefly, 100 μL of the MTT labeling reagent (final concentration, 0.5 mg/mL; Sigma-Aldrich) was added to each well for 3 h. Then, the supernatant was removed and DMSO was then added to allow the reduction of MTT into formazan crystals formed by metabolically active cells. The optical density of each well sample was determined at 570 nm using a microplate reader (VICTOR Nivo, Revvity). For each independent experiment, blank absorbance was determined from wells containing medium and MTT reagent but no cells, and the corresponding value was subtracted from all measurements. The average absorbance values from peptide-treated cells were normalized against control and cell viability was expressed as a percentage of the control. Cell viability assays were performed as a single experiment with three technical replicates (*n* = 3), and data are reported as mean ± standard deviation (SD).

### Statistical Analysis

In ThT fluorescence and MTT assays, data were obtained from three independent experiments (*n* = 3) and are reported as mean ± standard deviation (SD); error bars represent the SD of the three independent replicates. For SEM measurements, fibril diameters were determined from micrographs by performing repeated measurements along the same fibril using image analysis software. Fibril lengths, on the other hand, were calculated as the sum of individual connected segments. In both cases, the reported values represent the mean and the associated measurement uncertainty. The statistical significance between groups in cytotoxicity assays was assessed using one-way analysis of variance (ANOVA) followed by Bonferroni’s post hoc test for multiple comparisons, with *p* ≤ 0.05 considered significant. Analyses were performed using GraphPad Prism 8.0 (GraphPad Software, San Diego, CA).

## Results and Discussion

### Aggregation Behavior of NPM1 Fragments Monitored by ThT Fluorescence

The amino acid sequences of the NPM1_259–280_ and NPM1_263–280_ peptides, along with their calculated isoelectric points (pI) and estimated net charges at neutral pH, are reported in Figure 1A. Both fragments display identical theoretical pI values (10.02) and net charges (+1.9), indicating comparable overall electrostatic properties. The aggregation propensity of the peptides was monitored by using ThT fluorescence assays in the absence and presence of TFE 50% (v/v) (Figure 1B).

**Figure 1:**
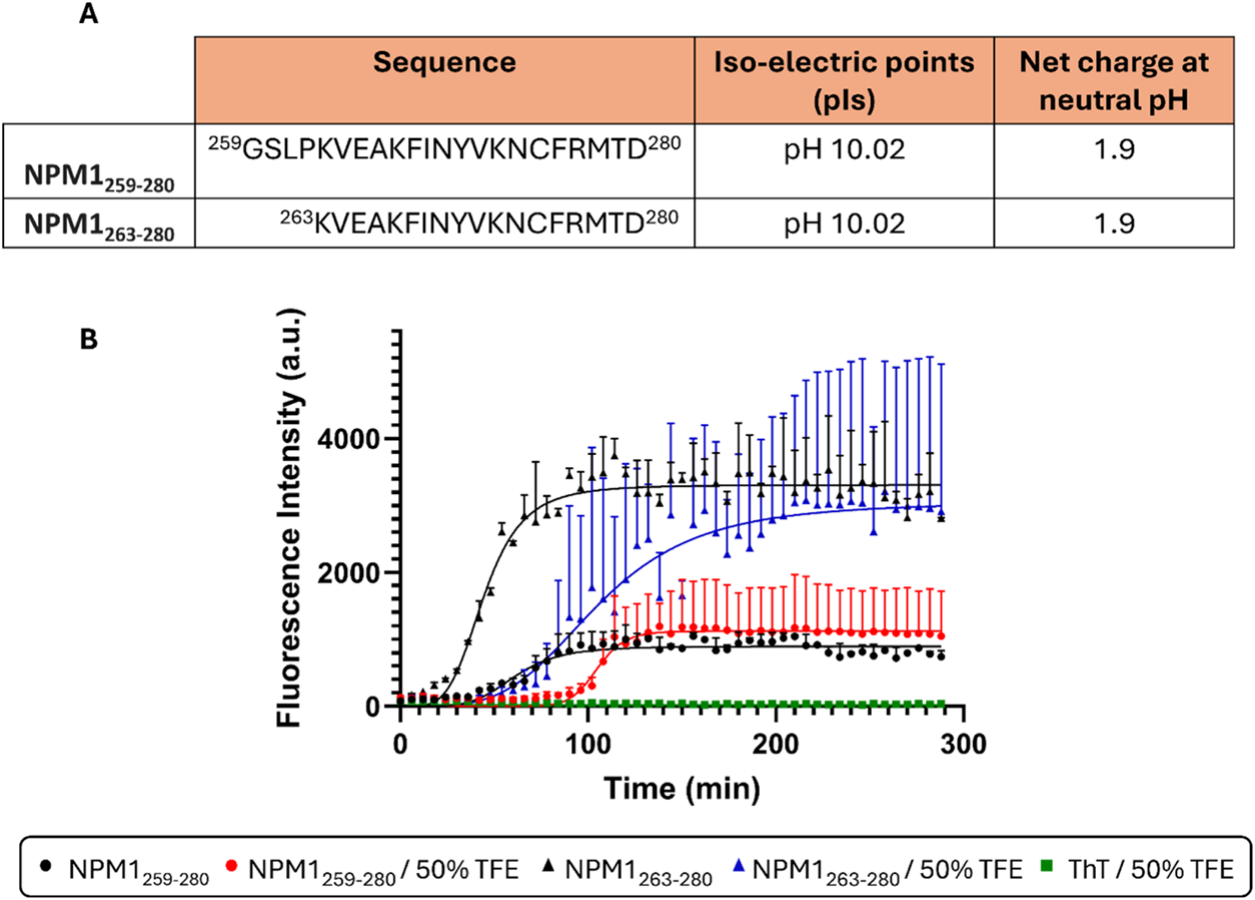
(A) NPM1_259–280_ and NPM1_263–280_ peptides analyzed in this study: sequences, pIs, and estimated net charges. (B) Kinetics of aggregation by ThT fluorescence assay of: NPM1_259–280_ (black circle), NPM1_259–280_ in the presence of TFE (red circle), NPM1_263–280_ (black triangle), and NPM1_263–280_ in the presence of TFE (blue triangle). ThT (50 μM) in the presence of TFE (green square) was evaluated as reference. Data represent the mean ± SD of three independent replicates.

In aqueous buffer, the two fragments showed markedly different aggregation kinetics. NPM1_263–280_ displayed the fastest and strongest aggregation response, yielding the lowest *t*_1/2_ value (≈46 min) and the highest maximum fluorescence intensity (Table 1). In contrast, NPM1_259–280_ appeared less prone to aggregation, exhibiting a *t*_1/2_ approximately twice as long and a more than 2-fold lower maximum intensity. For both peptides, the addition of TFE markedly delayed ThT fluorescence enhancement. This effect was most pronounced for the shortest fragment, for which the *t*_1/2_ increased 2-fold relative to aqueous conditions. Control experiments with ThT alone confirmed that the fluorescence changes originated from peptide aggregation. Because both fragments possess identical pI and net charge, their distinct aggregation behaviors cannot be attributed to electrostatic differences.

**Table 1:** Experimental Values of *t*_1/2_ and Maximum Fluorescence Intensity of NPM1_259–280_ and NPM1_263–280_ Peptides Both in the Absence and in the Presence of TFE^a^

samples	*t*_1/2_ (min)	maximum intensity (au) × 10^3^
NPM1_259–280_	61 ± 4	1.02 ± 0.06
NPM1_259–280_-TFE	105 ± 7	1.19 ± 0.08
NPM1_263–280_	46 ± 3	3.8 ± 0.3
NPM1_263–280_-TFE	98 ± 6	3.0 ± 0.2

a Values are reported as mean ± SD.

### Secondary Structure Evolution Monitored by CD, FT-IR, and NMR Spectroscopy

Circular dichroism (CD) and FT-IR spectroscopies were used to examine structural features associated with aggregation, both in the presence and absence of TFE. CD deconvolution data are provided in Table S1. In aqueous solution, both peptides displayed spectra with a single minimum at ∼202 nm (Figure 2A,C), consistent with predominantly disordered conformations, although a shoulder at ∼222 nm, more pronounced for NPM1_259–280_, was also observed. Over time, both sequences exhibited a progressive loss of Cotton effect due to aggregation.^54^ The Cotton effect is the differential absorption of left- and right-circularly polarized light by a chiral chromophore in the region of an electronic transition, observed as a characteristic positive or negative signal in circular dichroism spectra and can indicate canonical secondary structures.^55^

**Figure 2:**
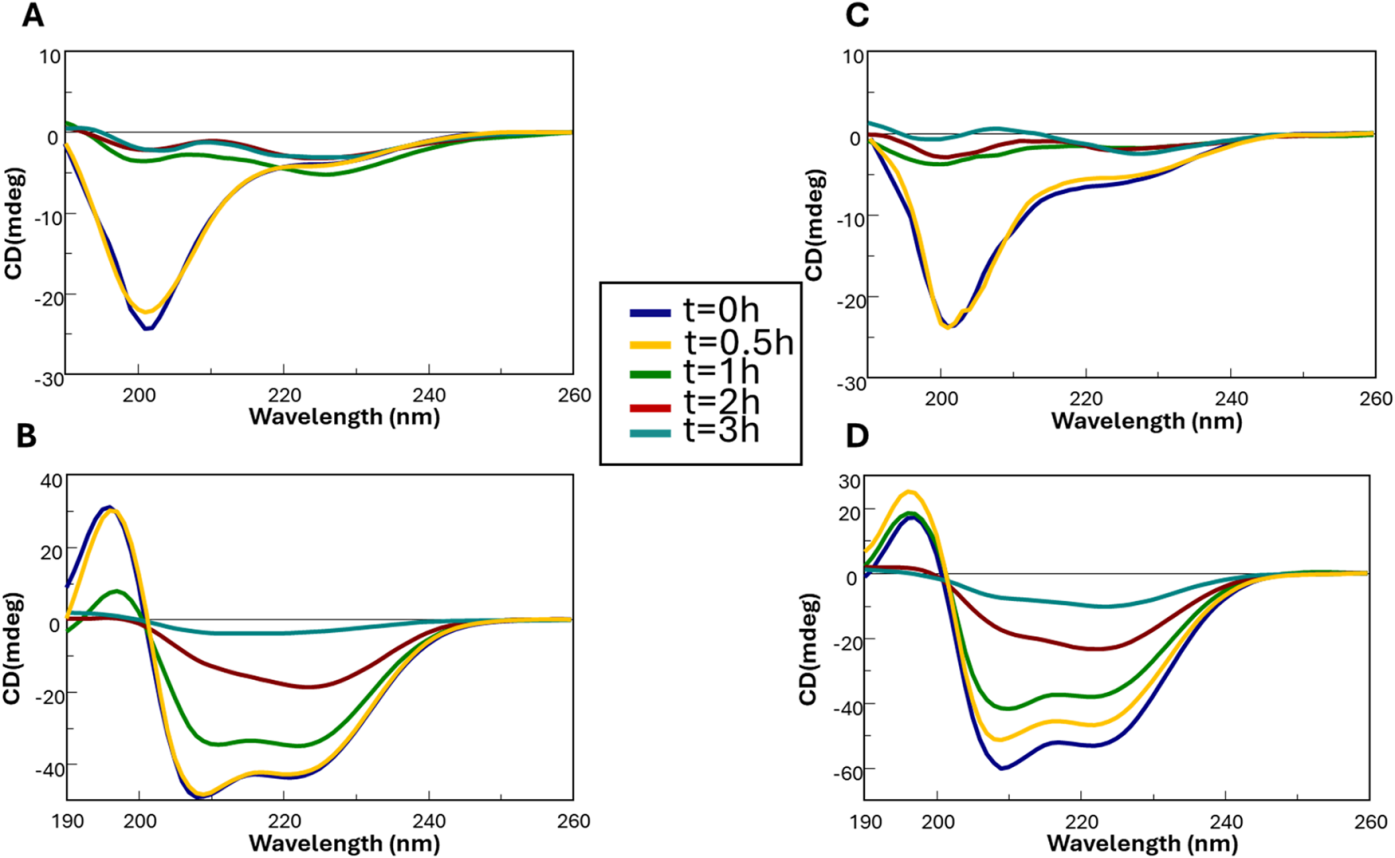
Overlay of CD spectra over time of: NPM1_259–280_ (A, B) and NPM1_263–280_ (C, D) in buffer, in the absence (A–C) and in the presence (B–D) of TFE.

In the presence of TFE (Figure 2B,D), both peptides showed increased ellipticity at 222 nm and a shift of the absolute minimum to ∼210 nm, indicative of a transition toward α-helical or otherwise more ordered conformations (Table S1). Time-dependent spectral changes confirmed a protective effect of helix formation, as the decrease in Cotton effect proceeded significantly more slowly than in aqueous buffer. Consistently, the evolution of CD ratio (minimum/222 nm) and Δλ_min_ values (the difference between the λ of absolute minimum at each time and the same value at *t* = 0, λ_*t_i_*_ – λ_*t*_0__) (Figure S1) further supports stabilization of α-helical structures in TFE. FT-IR spectra and deconvolution analyses (Figure S2) corroborated these findings: in TFE, a more pronounced band at ∼1658 cm^–1^ indicated enhanced helical content.

To further characterize the structural features of the two peptides, 1D ^1^H and 2D ^1^H–^1^H NMR experiments were performed. Both peptides appeared highly disordered in purely aqueous buffer, while in the presence of TFE (50%, v/v) secondary structure tendencies were evident.^56^ NMR spectra acquired at two peptide concentrations (200 and 640 μM; Figure S3) showed only minor concentration-dependent chemical shift variations, consistent with a TFE stabilizing effect of the predominant conformation. Spectra collected after 3 and 4 h (Figure S4) revealed no major time-dependent changes, reflecting a suppression of aggregation due to the presence of TFE. NMR spectra indicate conformational stabilization under conditions that correlate with delayed aggregation in ThT assays.

The 2D TOCSY and NOESY spectra (Figure 3) were characteristic of partially ordered peptides. Resonance assignments were achieved primarily through TOCSY and supported by NOESY correlations;^49^ however, spectral overlap and chemical shift degeneracy prevented unambiguous assignment of several spin systems (Tables S2 and S3).

**Figure 3:**
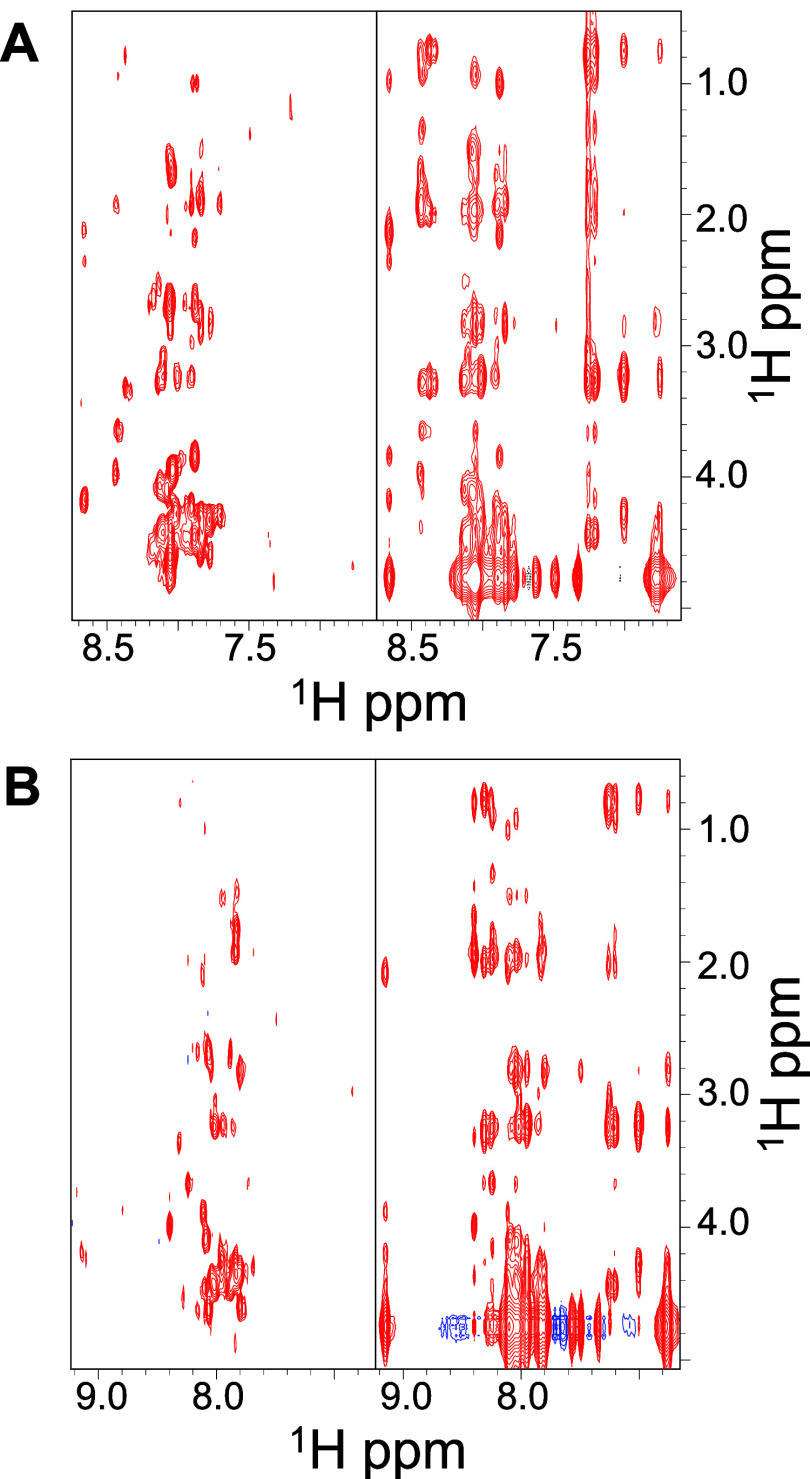
Comparison of 2D [^1^H–^1^H] TOCSY (left panels) and NOESY 300 (right panels) spectra acquired for NPM1_259–280_ (A) and NPM1_263–280_ (B) peptides (at 640 μM, in 10 mM sodium phosphate/TFE-*d*_3_ (50:50, v/v)). The HN/aromatic–aliphatic regions are shown.

Both peptides exhibited negative Hα chemical shift deviations (ΔδHα) from random-coil values (Figure 4A,C), indicating the presence of helical structure. A continuous stretch with |ΔδHα| > 0.1 ppm suggests that helicity spans residues K267–K273.^57,58^ The helical content estimated from ΔδHα values was 54% for both peptides. The NOE patterns (Figure 4B,D) further support the presence of helical segments: characteristic short- and medium-range NOEs, including Hα*_i_*–Hβ_*i*+3_ and Hα*_i_*–HN_*i*+3_ interactions, were observed from residues K263 to K273. The helical region identified by ΔδHα (K267–K273) overlaps with the amyloidogenic segment previously reported for NPM1, suggesting that helix formation competes with β-sheet organization.

**Figure 4:**
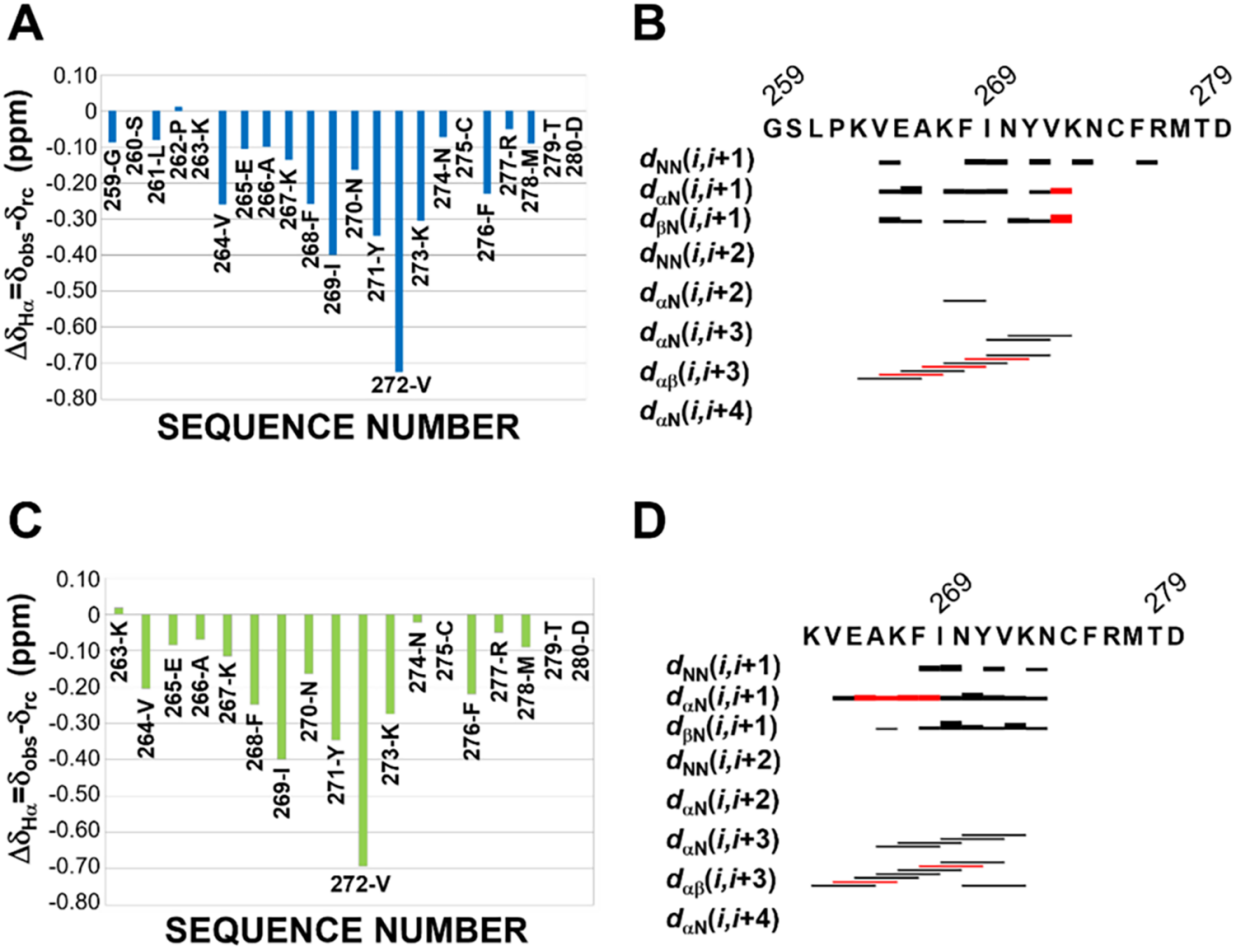
Secondary structure analyses by NMR of NPM1_259–280_ (A, B) and NPM1_263–280_ (C, D) (at 640 μM concentration in 10 mM sodium phosphate/TFE-*d*_3_ (50:50, v/v)). In (A) and (C), chemical shift deviations of observed Hα proton with respect to random-coil values (ΔδHα) are reported. ΔδHα are set equal to 0 when involving ambiguous Hα assignments. In (B) and (D), the peptide NOE patterns are reported; red bars indicate ambiguous NOEs due to spectral overlaps.

The shorter peptide, NPM1_263–280_, exhibited a slightly larger number of helical NOEs than NPM1_259–280_ (Table S4).

### Morphological Characterization by Scanning Electron Microscopy

SEM imaging of aggregates formed after 4 h of stirring revealed notable morphological differences between the two NPM1 fragments (Figure 5). In aqueous solution, the longer peptide, NPM1_259–280_, formed shorter but substantially thicker fibrils than the shorter fragment NPM1_263–280_ (Figure 5A,C; Table 2). In the presence of TFE (Figure 5B,D), fibril diameter remained largely unchanged; however, fibril length increased and distinct helical twists along the fibril axis became apparent (Table 2).

**Figure 5:**
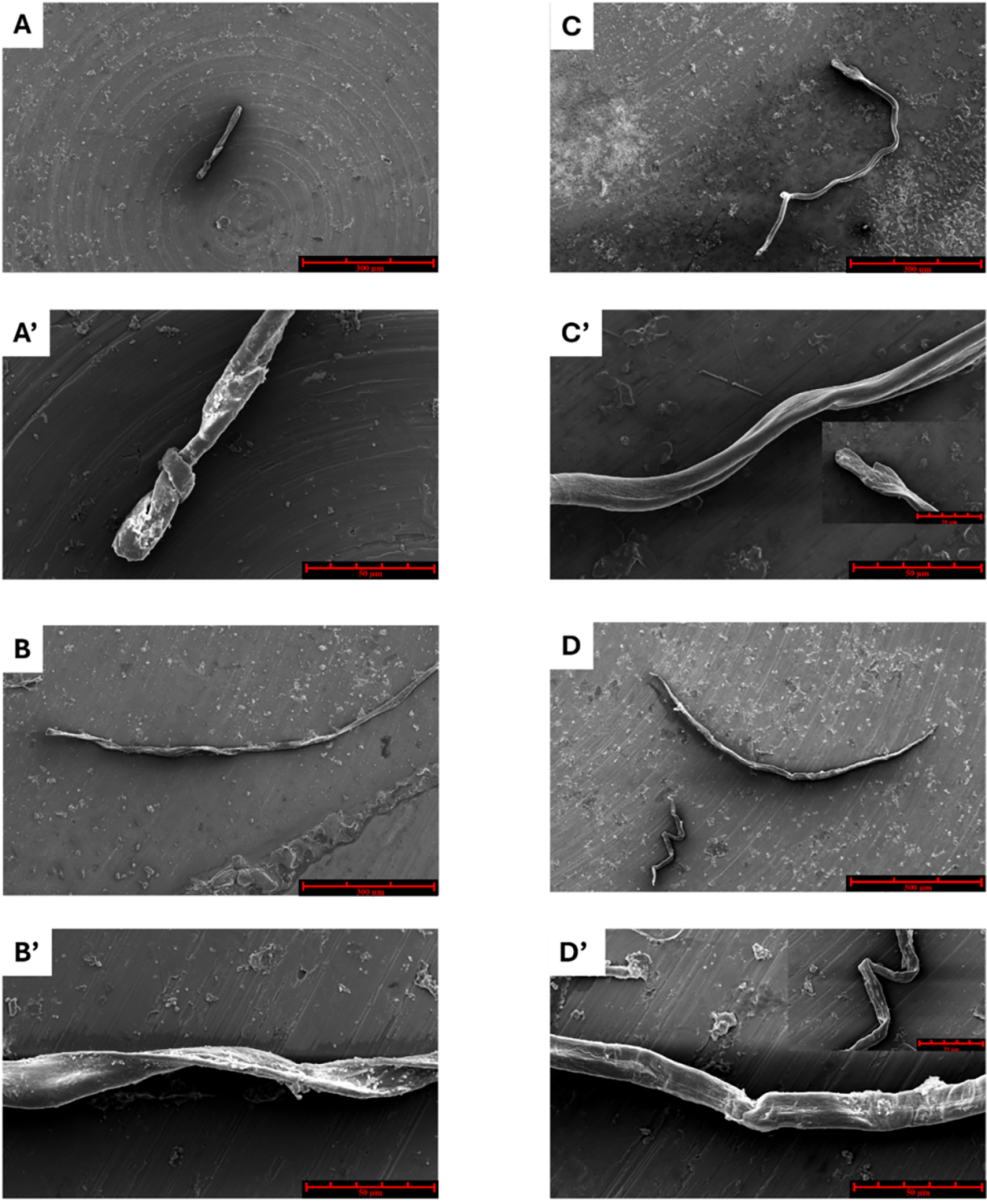
SEM micrographs after 4 h of aggregation of: NPM1_259–280_ (A, A′) (B, B′) and NPM1_263–280_ (C, C′) (D, D′) in buffer, in the absence (A–C) and in the presence (B–D) of TFE (50%, v/v). Overviews of the surface of samples at 300 μm (A–D) and 50 μm (A′–D′). Zooms of the rest of the fiber (C′) and the second fiber present (D′) are reported as inset.

**Table 2:** SEM Analysis^a^

samples	length (μm)	average diameter (μm)	knots presence	twists number
NPM1_259–280_	231.200 ± 0.010	15 ± 2	yes	2
NPM1_259–280_-TFE	720.40 ± 0.02	14 ± 5	no	6
NPM1_263–280_	842.50 ± 0.04	12 ± 3	no	3
NPM1_263–280_-TFE	first 961.70 ± 0.03	first 16 ± 4	no	first 6
	second 54.100 ± 0.010	second 12 ± 3		second 4

a The average fiber diameter and length, as well as the presence of knots and the number of twists in the fibers obtained for NPM1_259–280_ and NPM1_263–280_ peptides both in the absence and in the presence of TFE (50%, v/v).

Higher-magnification imaging (Figure S5) provided additional insights into fibril surface organization. In the absence of TFE, both peptides formed heterogeneous fibrils with rough surfaces, frequent branching points, and occasional smoother sections, while, in TFE, fibrils appeared more compact, with NPM1_263–280_ in particular forming tightly aligned bundles with clearly discernible axial undulations.^59^

### Cellular Cytotoxicity of NPM1 Fragments

The potential cytotoxicity of the peptides was assessed in OCI-AML2 cells using an MTT viability assay, in both the absence and presence of TFE. TFE was not intended for translational or physiological interpretation; cytotoxicity assays were performed solely to assess whether aggregation state correlates with acute cellular responses under comparative conditions. As shown in Figure 6A, both NPM1_259–280_ and NPM1_263–280_ induced a time-dependent decrease in cell viability after 4 h of incubation, with overall comparable effects. In contrast, in the presence of TFE (Figure 6B), they did not affect cell viability under the same conditions.

**Figure 6:**
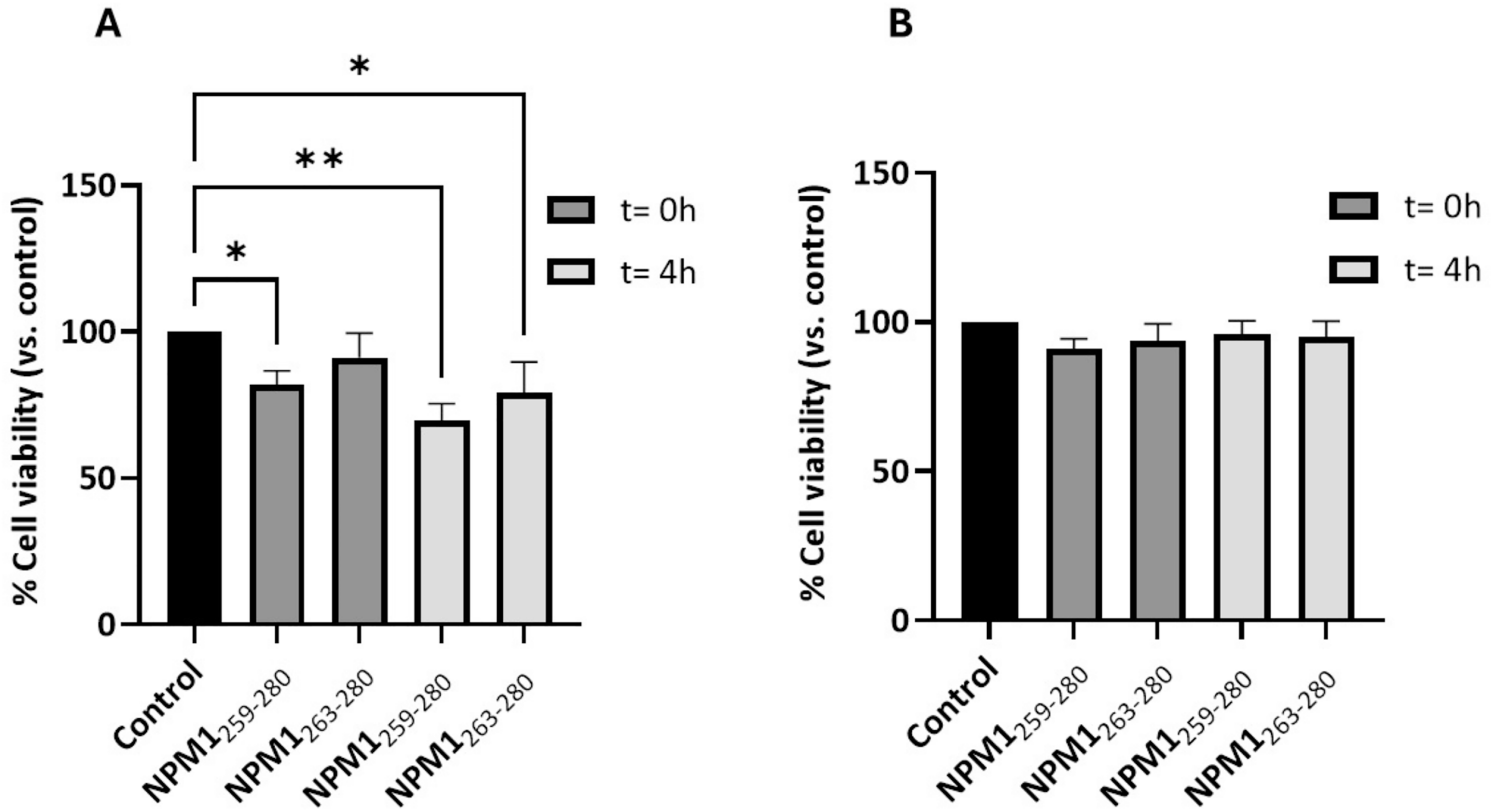
Cell viability effects of NPM1_259–280_ and NPM1_263–280_ in the absence (A) and in the presence (B) of TFE (50%, v/v). The histograms report the values of percentage cell viability (vs CTRL) using OCI-AML2 cells at two different times: *t* = 0 and 4 h. Data are from a single experiment performed with three technical replicates (*n* = 3) and are reported as mean ± SD. The statistical analysis was performed by GraphPad Prism 10 software using two-way ANOVA corrected for multiple comparison by the Dunnett test (*p < 0.05, **p < 0.01).

## Conclusions

In this study, we performed a comparative biophysical and cellular analysis of two closely related NPM1 C-terminal fragments, NPM1_259–280_ and NPM1_263–280_, to investigate how minimal N-terminal sequence extensions influence conformational behavior, aggregation propensity, and cellular cytotoxicity.

Although the two peptides share identical theoretical electrostatic properties, they exhibited markedly different aggregation kinetics, structural features, and aggregate morphologies, underscoring the importance of the local sequence context in modulating amyloid formation.

ThT fluorescence assays revealed that the shorter fragment, NPM1_263–280_, displayed faster aggregation kinetics and higher ThT fluorescence intensity than NPM1_259–280_, suggesting that the four-residue N-terminal extension (GSLP) in the longer peptide modulates aggregation efficiency, possibly by affecting early conformational sampling or sterically interfering with β-sheet organization. Consistent with this interpretation, spectroscopic analyses by CD, FT-IR, and NMR showed that both peptides possess an intrinsic propensity to adopt α-helical conformations, particularly within residues 267–273, a region overlapping with the previously identified amyloidogenic segment. The stabilization of helical conformations by TFE correlated with delayed aggregation kinetics and reduced ThT signal development, supporting an inverse relationship between helical content and rapid β-rich assembly.

Morphological characterization by SEM further demonstrated that the two fragments form fibrillary aggregates with distinct dimensions and surface features, indicating that even short-sequence extensions influence not only aggregation kinetics but also the architecture of the resulting assemblies. In the presence of TFE, fibril organization and bundling were altered, consistent with changes in aggregation pathways rather than the complete suppression of aggregate formation.

Functionally, both peptides reduced cell viability in OCI-AML2 cells under aggregation-prone conditions, whereas treatments performed in the presence of TFE did not produce detectable cytotoxic effects. Despite the distinct fibril morphologies, the current data do not allow us to establish a direct correlation between the fibril architecture and effects on cell viability.

Although this study uses nonphysiological conditions to probe intrinsic conformational preferences and their relationship to aggregation, its aim is to uncover fundamental principles. Future work should therefore test the generality of this protective effect under physiologically relevant conditions, such as near-physiological salt concentrations, low micromolar peptide concentrations, and the presence of molecular chaperones or cellular crowding agents.

Overall, our results highlight how minimal N-terminal sequence variations can significantly modulate the conformational landscape and aggregation behavior of fragment 264–277 of NPM1. Rather than defining a specific mechanistic pathway, this work emphasizes the role of transient secondary structure propensities and local sequence context in shaping aggregation outcomes. These findings contribute to a broader understanding of NPM1 misfolding behavior and provide a framework for future studies aimed at dissecting how subtle sequence features influence protein aggregation in disease-relevant systems.

## Supplementary Material



## Notes

Additional CD, FT-IR, NMR, and SEM analyses of NPM1 fragments; CD kinetic parameters, FT-IR spectra and second-derivative analyses, concentration- and time-dependent ^1^H NMR spectra, and SEM micrographs (Figures S1–S5); and CD spectral deconvolution, ^1^H chemical shift assignments, and comparison of α-helical NOEs (Tables S1–S4) (PDF)

## Abbreviations

AD: Alzheimer’s disease
AML: acute myeloid leukemia
CD: circular dichroism
FT-IR: Fourier transform infrared spectroscopy
HFIP: 1,1,1,3,3,3-hexafluoro-2-propanol
LLPS: liquid–liquid phase separation
MTT: 3-(4,5-dimethylthiazol-2-yl)-2,5-diphenyltetrazolium bromide
NOESY: nuclear Overhauser enhancement spectroscopy
NPM1: nucleophosmin 1
PD: Parkinson’s disease
SEM: scanning electron microscopy
SSPS: solid-state peptide synthesis
TFE: 2,2,2-trifluoroethanol
ThT: thioflavin T
TOCSY: total correlation spectroscopy
