# Supplementary material for: Helix-Aggregation Interplay in Nucleophosmin 1: Structural, Morphological, and Cytotoxic Consequences of Fragment Length
Source: ACS Omega. 2026 May 13;11(20):30169–78. doi: 10.1021/acsomega.6c03067 (PMC13216914; doi:10.1021/acsomega.6c03067)
Supplement: Supplementary file 1 [file ao6c03067_si_001.pdf]

SUPPORTING INFORMATION

Helix–Aggregation Interplay in Nucleophosmin 1: Structural, Morphological, and Cytotoxic Consequences of Fragment Length

Daniele Florio[1], Ilaria Leone[1], Sara La Manna[2], Alessia Cugudda[2], Flavia Anna Mercurio[3], Marilisa Leone[3] and Daniela Marasco[2*]

[1]IRCCS SYNLAB SDN, via G. Ferraris 144, 80146, Naples, Italy

[2]Department of Pharmacy, School of Medicine and Surgery, University of Naples Federico II, Via Domenico Montesano 49, 80131, Naples, Italy.

[3]Institute of Biostructures and Bioimaging (IBB), CNR, Via P. Castellino 111, Naples 80131, Italy.

*Corresponding author

List of Supplementary Material

Figure S1. NPM1$_{259-280}$ (red circle) and NPM1$_{263-280}$ (blue square) in the presence of 50% TFE: (A) *CD* ratio *versus* time and (B) $\Delta\lambda_{min}$ (λ_{ti}- λ_{t0}) *versus* time. The value at t=3h were not evaluated since Cd signal was too low.

Figure S2. FT-IR spectra and secondary-derivative analysis of: NPM1$_{259-280}$ (A, C) and NPM1$_{263-280}$ (B, D) in buffer, in the absence (green) and in the presence (blue) of 50% TFE after 15 minutes of aggregation.

Figure S3. Comparison of 1D [^{1}H] NMR spectra acquired at different concentrations of NPM1$_{259-280}$ (A) and NPM1$_{263-280}$(B) peptides. blue and red trace refer to 640 µM and 200 µM, respectively. All spectra were acquired in 10 mM sodium phosphate/TFE (50/50, v/v) and T=25°C.

Figure S4. Comparison of 1D [^{1}H] NMR spectra of NPM1$_{259-280}$ (A) and NPM1$_{263-280}$ (B) peptides at different time points: red for t =0 (freshly dissolved), blue and green spectra refer to t=3h and t =4h, respectively, at 640 µM in 10 mM sodium phosphate/TFE (50/50, v/v)).

Figure S5. SEM micrographs after 4 h of stirring of: NPM1$_{259-280}$ (A, B) and NPM1$_{263-280}$(C, D) in buffer, in the absence (A-C) and in the presence (B-D) of 50% TFE. Overviews of the surface of samples at 3 µm.

Table S1. Deconvolution of CD spectra of NPM1 fragments at indicates times, in the absence and in the presence of 50% TFE.

Table S2. ^{1}H chemical shifts of NPM1$_{259-280}$ peptide (640 µM, in 10 mM NaP/TFE (50/50, v/v)), T=25°C and pH=7.45. Ambiguous assignments are marked in red. N.D. stands for “not determined”.

Table S3. ^{1}H chemical shifts of $NPM1_{263-280}$ peptide (640 µM, in 10 mM NaP/TFE (50/50, v/v), T=25°C and pH=7.32. Ambiguous assignments are marked in red. N.D. stands for not determined.

Table S4. Comparison of distinctive α-helical NOEs found in the NOESY spectra of $NPM1_{259-280}$ and $NPM1_{263-280}$ peptides. NOEs in red are ambiguous.

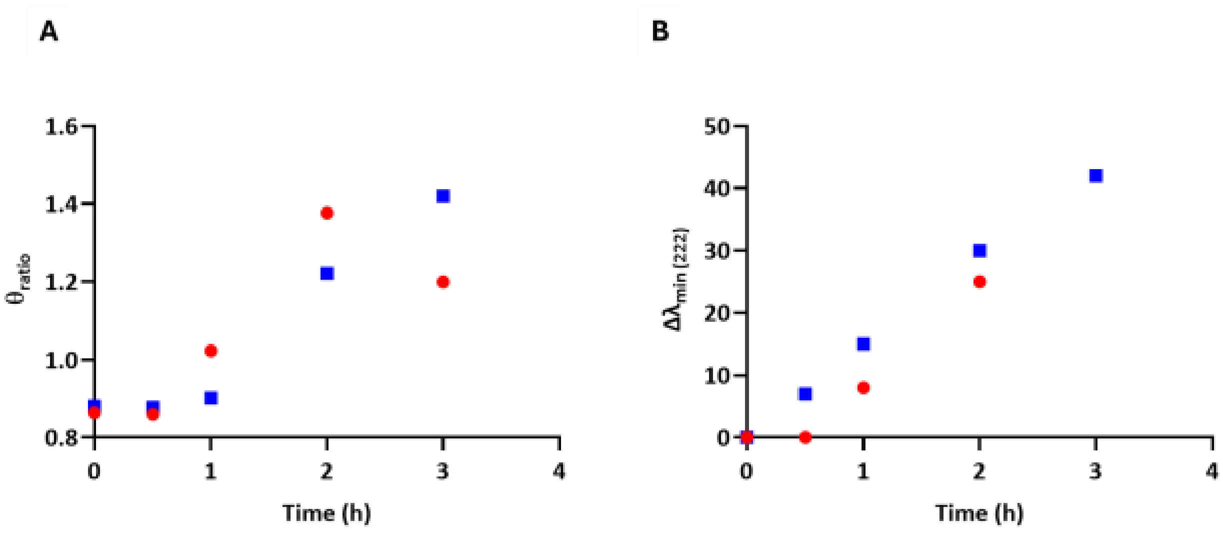

Figure S1. NPM1$_{259\text{-}280}$ (red circle) and NPM1$_{263\text{-}280}$ (blue square) in the presence of 50% TFE: (A) *CD* ratio *versus* time and (B) $\Delta\lambda_{min}$ (λ_{ti}- λ_{t0}) *versus* time. The value at t=3h were not evaluated since Cd signal was too low.

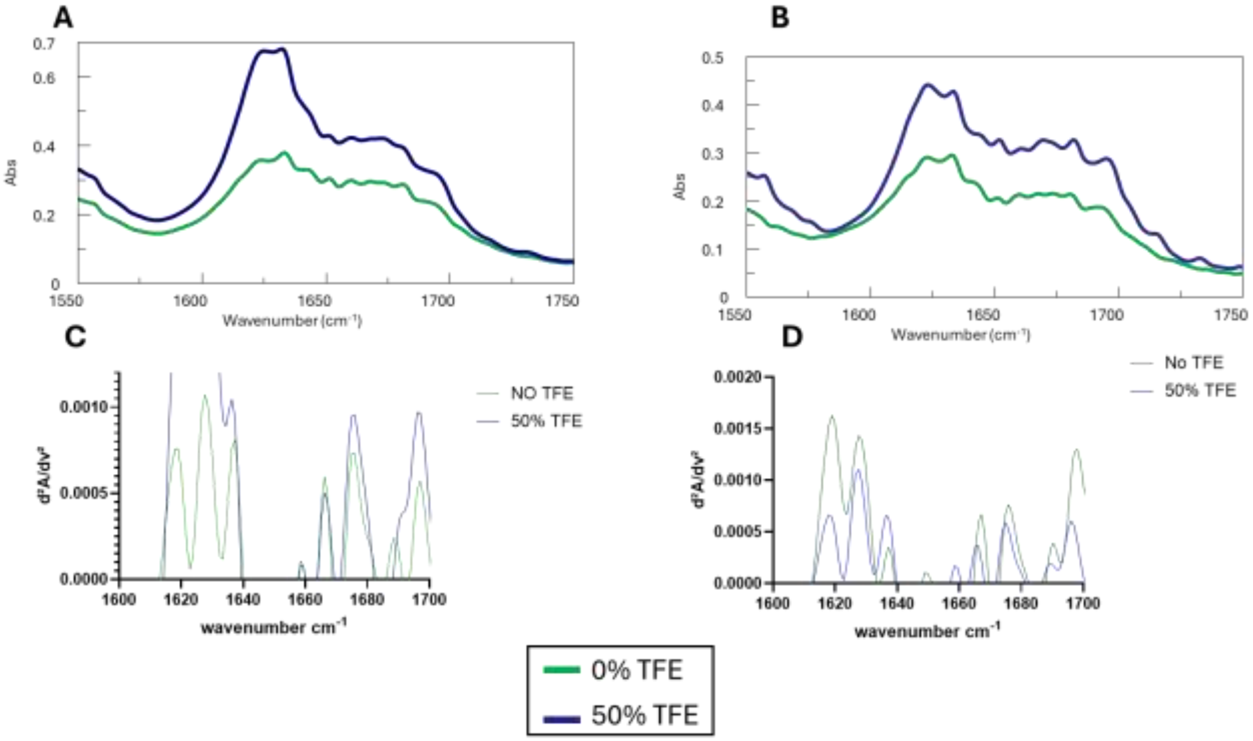

Figure S2. FT-IR spectra and secondary-derivative analysis of: NPM1$_{259-280}$ (A, C) and NPM1$_{263-280}$ (B, D) in buffer, in the absence (green) and in the presence (blue) of 50% TFE after 15 minutes of aggregation.

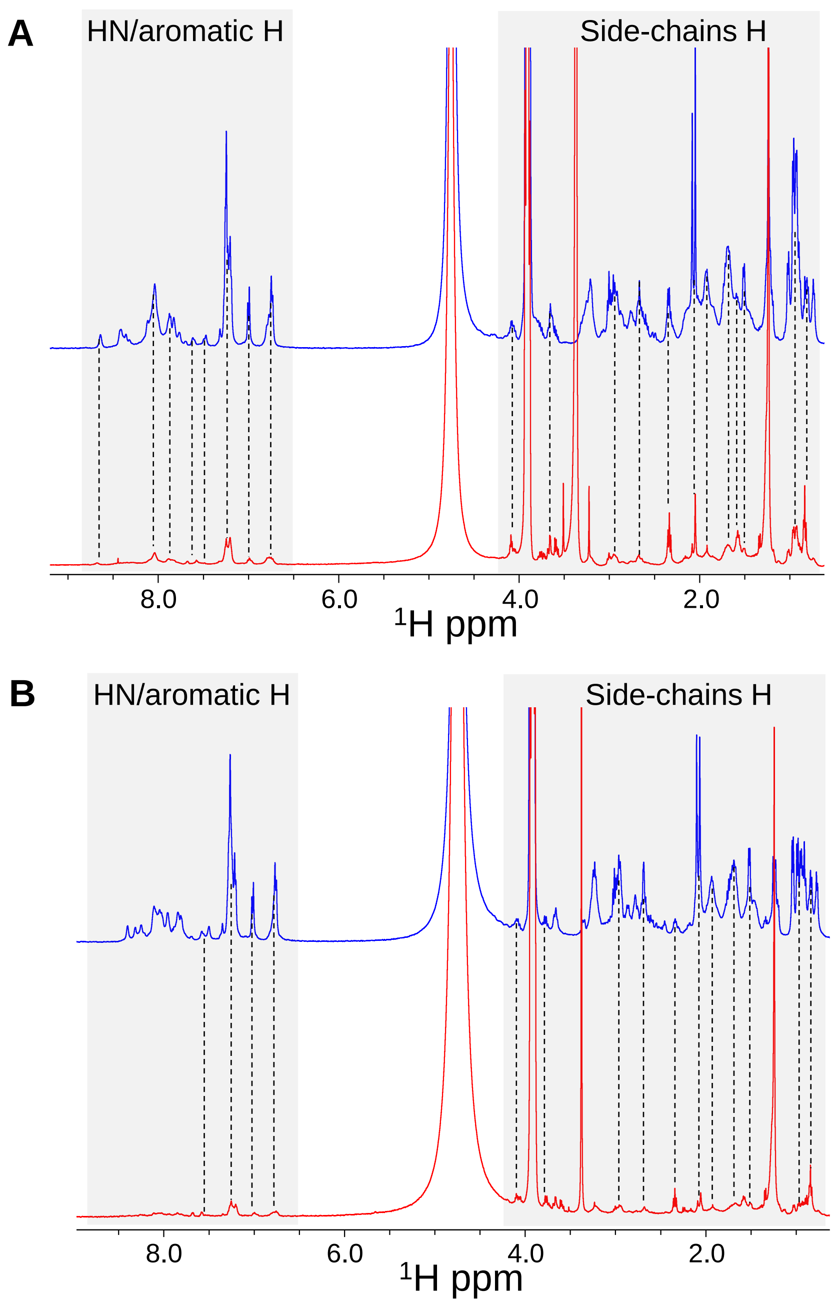

Figure S3. Comparison of 1D [^{1}H] NMR spectra acquired at different concentrations of NPM1$_{259\text{-}280}$ (A) and NPM1$_{263\text{-}280}$(B) peptides. blue and red trace refer to 640 μM and 200 μM, respectively. All spectra were acquired in 10 mM sodium phosphate/TFE (50/50, v/v) and T=25°C.

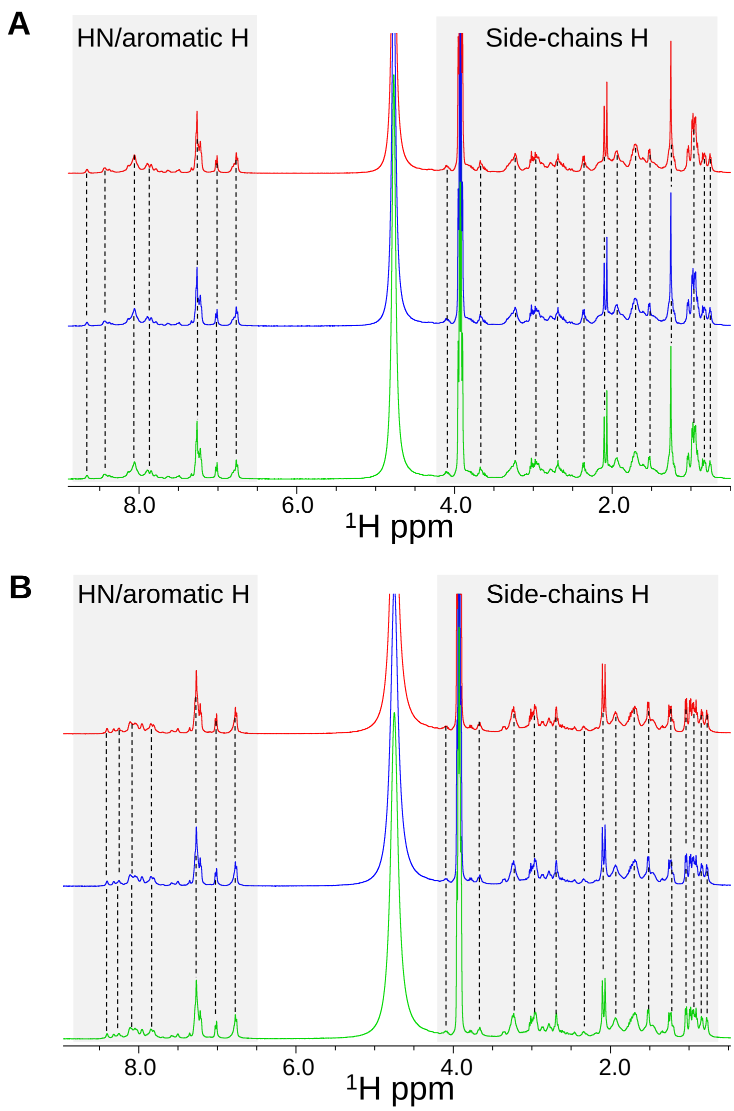

Figure S4. Comparison of 1D [^{1}H] NMR spectra of NPM1$_{259-280}$ (A) and NPM1$_{263-280}$ (B) peptides at different time points: red for t =0 (freshly dissolved), blue and green spectra refer to t=3h and t =4h, respectively, at 640 μM in 10 mM sodium phosphate/TFE (50/50, v/v))

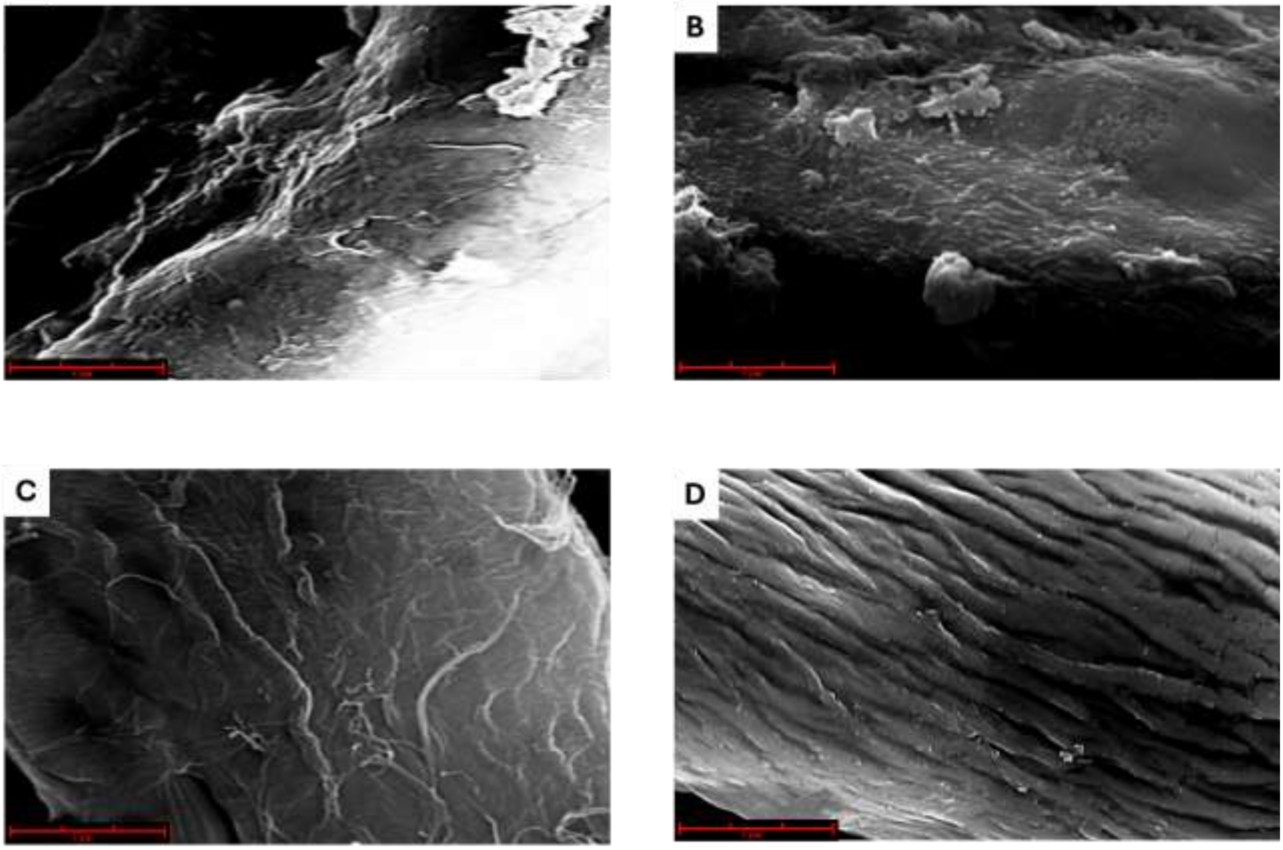

Figure S5. SEM micrographs after 4 h of stirring of: NPM1$_{259-280}$ (A, B) and NPM1$_{263-280}$ (C, D) in buffer, in the absence (A-C) and in the presence (B-D) of 50% TFE. Overviews of the surface of samples at 3 μm.

Table S1. Deconvolution of CD spectra of NPM1 fragments at indicates times, in the absence and in the presence of 50% TFE.

$NPM1_{259-280}$	**HELIX**	**β-sheet**	**TURN**	**OTHERS**
0	3.6	25.5	16.1	54.8
0.5	3.3	28.2	16.9	51.6
1	0.0	32.5	14.9	52.7
2	0.0	36.4	14.1	49.5
	**HELIX**	**β-sheet**	**TURN**	**OTHERS**
$NPM1_{259-280}$ -TFE				
0	49.8	15.3	8.4	26.5
0.5	50.4	15.9	7.3	26.5
1	29.1	26.0	10.8	34.2
2	10.9	29.7	14.0	45.3
	**HELIX**	**β-sheet**	**TURN**	**OTHERS**
$NPM1_{263-280}$				
0	5.0	24.5	16.3	54.1
0.5	6.7	15.3	0.0	61.2
1	0.0	32.7	14.8	52.5
2	0.0	35.7	14.3	20.1
	**HELIX**	**β-sheet**	**TURN**	**OTHERS**
$NPM1_{263-280}$ -TFE				
0	67.4	32.6	0.0	0.0
0.5	70.5	24.8	4.7	0.0
1	55.7	21.2	9.1	14.0
2	15.8	32.0	11.4	40.7

Table S2. [1]H chemical shifts of NPM1$_{259-280}$ peptide (640 µM, in 10 mM NaP/TFE (50/50, v/v)), T=25°C and pH=7.45. Ambiguous assignments are marked in red. N.D. stands for "not determined".

	HN	Hα	Hβ	Hγ	Other
259-G	8.04	3.94			
260-S	N.D.	4.10	3.61-3.67		
261-L	8.05	4.60	1.61-1.72		δCH3 0.94-0.97
262-P		4.45	2.05	1.95-2.31	Hδ 3.67-3.80
263-K	7.84	4.31	1.93	1.50	Hδ 1.75<br>Hε 3.00
264-V	7.88	3.84	2.16	γCH3<br>0.99-1.05	
265-E	8.66	4.16	2.11	2.36	
266-A	8.07	4.13	1.52		
267-K	8.08	4.08	1.97	1.45	Hδ 1.69<br>Hε 2.95
268-F	8.14	4.43	3.26		Hδ 7.20<br>Hε 7.27
269-I	8.42	3.66	1.97	γCH3 0.95<br>1.33-1.82	δCH3 0.91
270-N	8.05	4.39	2.79-2.86		Hδ<br>6.79-7.50
271-Y	8.01	4.28	3.23		Hδ 7.01<br>Hε 6.76
272-V	8.37	3.32	2.02	γCH3	

				0.75-0.82	
273-K	8.43	3.97	1.90	1.41	Hδ 1.67<br>Hε 2.95
274-N	7.84	4.53	2.75-2.87		
275-C	7.88	4.45	2.69		
276-F	8.11	4.43	3.08-3.23		Hδ 7.26<br>Hε 7.21
277-R	7.91	4.27	1.92	1.67-1.74	Hδ 3.23
278-M	N.D.	4.45	2.09-2.18	2.61-2.69	
279-T	7.78	4.36	4.32	γCH3 1.21	
280-D	8.06	4.60	2.68		

Table S3. [1]H chemical shifts of NPM1$_{263-280}$ peptide (640 μM, in 10 mM NaP/TFE (50/50, v/v), T=25°C and pH=7.32. Ambiguous assignments are marked in red. N.D. stands for not determined.

	HN	Hα	Hβ	Hγ	Other
263-K	7.83	4.37	1.93	1.49	Hδ 1.74<br>Hε 3.01
264-V	8.11	3.89	2.09	γCH3<br>0.98-1.04	
265-E	9.14	4.18	2.08	2.33-2.45	
266-A	7.97	4.16	1.51		
267-K	8.10	4.10	1.94	1.48	Hδ 1.69<br>Hε 2.95
268-F	8.02	4.44	3.25		Hδ 7.20<br>Hε 7.27
269-I	8.24	3.66	1.96	γCH3 0.95	δCH3 0.90

				1.32-1.79	
270-N	8.05	4.39	2.79-2.86		Hδ 6.76-7.50
271-Y	7.95	4.28	3.21		Hδ 7.01 Hε 6.76
272-V	8.32	3.35	2.01	γCH3 0.76-0.84	
273-K	8.40	4.00	1.91	1.45	Hδ 1.67 Hε 2.95
274-N	7.80	4.58	2.79-2.86		
275-C	7.88	4.42	2.69		
276-F	8.02	4.44	3.02-3.22		Hδ 7.26 Hε 7.21
277-R	7.85	4.27	1.92	1.65	Hδ 3.23
278-M	8.11	4.45	2.10-2.17	2.61	
279-T	7.80	4.31	4.36	γCH3 1.23	
280-D	8.07	4.61	2.68		

Table S4. Comparison of distinctive α-helical NOEs found in the NOESY spectra of NPM1$_{259\text{-}280}$ and NPM1$_{263\text{-}280}$ peptides. NOEs in red are ambiguous.

	**NPM1$_{259\text{-}280}$**	**NPM1$_{263\text{-}280}$**
$d_{\alpha N}(i,i+3)$	K267-N270<br>I269-V272	A266-I269<br>K267-N270<br>I269-V272<br>N270-K273
$d_{\alpha\beta}(i,i+3)$	K263-A266<br>V264-K267<br>E265-F268<br>A266-I269<br>K267-N270<br>F268-Y271<br>I269-V272	K263-A266<br>V264-K267<br>E265-F268<br>A266-I269<br>K267-N270<br>F268-Y271<br>I269-V272<br>N270-K273
